# Does the ratio of stem‐to‐femur length matter? A validated finite element analysis

**DOI:** 10.1002/jeo2.70484

**Published:** 2025-10-31

**Authors:** Georgios Banis, Charoula Kousiatza, Maria Sarkiri, Κostas Touloupas, Alexis Spiliotopoulos, Panagiotis Kontopoulos, Styliani Stergiadou, Michael Hantes, Aristeidis Zibis, Konstantinos Malizos, Ermioni Pasiou, Stavros Kourkoulis, Konstantinos Kalkanis, Ilias Stavrakas, Athanasios Batagiannis

**Affiliations:** ^1^ Akmelogi SMPC Athens Greece; ^2^ Faculty of Medicine University of Thessaly Larisa Greece; ^3^ National Technical University of Athens School of Applied Mathematical and Physical Sciences Athens Greece; ^4^ Department of Electrical and Electronics Engineering University of West Attica Athens Greece; ^5^ 3 PSI Ltd. Larnaka Cyprus

**Keywords:** micromotion, personalized stem, stem length, stress‐shielding, total hip arthroplasty

## Abstract

**Purpose:**

Total hip arthroplasty is among the most successful orthopedic procedures; however, approximately 10%–20% of patients require revision surgery within 15–20 years. Potential failure mechanisms at the bone–implant interface include stress shielding and excessive micromotion. This experimental work aims at examining the optimal ratio of femur length and stem length with respect to primary stability and stress shielding effect, employing a validated finite element analysis.

**Methods:**

One hundred human femora were reconstructed from computed tomography (CT) scans, and for each femur, 13 personalized cementless stems were designed with varying lengths (70–130 mm, in 5 mm increments), resulting in 1300 finite element models. Each model was analyzed under standardized joint and muscle loading conditions. The model was validated experimentally after cadaveric implantation with digital image correlation (DIC) under static loading. Primary outcomes included micromotion at the bone–implant interface and proximal stress shielding. Optimal stem length was determined as the ratio of stem‐to‐femur length that minimized the combined normalized scores of micromotion and stress shielding. The effect of stem‐to‐femur length ratio was analysed with linear regression.

**Results:**

The analysis revealed a correlation between femoral length and stem length. Longer stems provided better initial stability and reduced micromotion. On the other hand, shorter stems demonstrated less proximal stress shielding and a better stress distribution resembling that of the intact femur. The optimal stem length was found to be 18.5% ± 2.7% (mean ± SD) of the femur length in terms of the mean of calculated micromotions and stress shielding, indicating that personalized stem length selection can enhance the bone–implant osseointegration.

**Conclusion:**

The selection of a stem length that corresponds proportionally to femoral length can improve primary stability and stress shielding upon implantation, highlighting the importance of enhancing initial fixation in order to extend the lifespan of hip replacements.

**Level of Evidence:**

N/A.

**Abbreviations**
3D: three‐dimensional
ADAM: atomic diffusion additive manufacturing
CT: computer tomography
DIC: digital image correlation
FEA: finite element analysis
FEM: finite element model
NN: neural network architecture
PMMA: poly(methyl methacrylate)
THA: total hip arthroplasty
UNETR: UNet Transformers

## INTRODUCTION

More than one million total hip arthroplasty (THA) operations take place annually worldwide, and this number is expected to rise in the coming years [12, 28]. Despite the universal acknowledgement of THA's success, 10%–20% of hip implants fail within 15–20 years, with aseptic loosening remaining a major cause of revision, thereby limiting their durability in younger patients who often require multiple revisions [4, 7, 12, 13, 28].

Metallic femoral stems alter the natural load transfer to the femur, leading to periprosthetic bone resorption according to Wolff's remodeling law [21, 22, 46], and eventually to implant loosening. This phenomenon, namely stress shielding, is more pronounced in the proximal femur, indicating the necessity of restoring the physiological proximal stress distribution [7, 12, 18, 19, 20, 21, 39, 45]. Stress shielding has several clinically important consequences. By reducing the physiological load in the proximal femur, it may contribute to aseptic loosening of the implant, although the extent of this role remains questioned [7, 20, 21, 45]. Furthermore, stress shielding is associated with decreased bone density and increased risk of periprosthetic fractures [39]. Finally, bone loss due to stress shielding complicates future revision surgeries, as reduced proximal bone stock makes reimplantation more challenging [39]. Another factor impeding the long‐term stability of the bone–implant system is the relative micromotion at the bone–implant interface in the first weeks following the operation. Micromotion exceeding 150 μm results in the formation of fibrous tissue, thus preventing osseointegration of the femoral stem and risking the stability of the artificial hip‐joint [12, 13, 18, 28, 32, 39, 46].

The aforementioned failure mechanisms have been extensively studied in recent decades. Short stems have been suggested as a potential solution since through the filling of the metaphyseal femur, proximal load transfer is fostered [25, 37]. However, they are prone to intraoperative misalignment or postoperative instability, including axial subsidence, which could result in altered biomechanics or even hip dislocation [16, 41]. The advent of cutting‐edge technologies, like Additive Manufacturing, Artificial Intelligence and Topology Optimization, has expanded the possibilities for custom implant design and manufacturing [18, 22, 36, 37].

Despite the customization trend as well as the plethora of publications looking into the value of shortening femoral stems [9, 14, 16, 27, 46], only a few observational studies have attempted to link the appropriate implant size to anthropometric characteristics. To the best of the authors’ knowledge, a single retrospective study implies a correlation between femoral stem size and shoe size [35]. Given that femoral bone length is proportional to an individual's height [6], it may also be hypothesized that femoral stem length directly correlates with femur length. Based on the available literature, few biomechanical studies have attempted to evaluate the stem length influence on stress patterns and initial stability [27, 46], without, however, suggesting an association with the femur length or another anthropometric index. In their study on cemented stems, *Li* et al. [27] found that short stem lengths could effectively enhance the proximal load in the femur without posing additional fracture risk. *Yamako* et al. [46] studied experimentally cementless short stems by analyzing strain distribution and concluded that with regard to a reduction in stem length, there is a trade‐off between stress shielding and initial stability. To address these questions, we employed a validated finite element analysis (FEA) approach, which allows systematic evaluation of implant biomechanics across a wide range of anatomies and stem lengths. The hypothesis of this study is that the optimal femoral stem length is directly proportional to the femur length, when trying to simultaneously minimize the stress shielding effect and the developed micromotion. This research aims to (i) develop and validate an FE model, (ii) study the performance of different lengths of personalized cementless stems with respect to primary stability and reduction of stress shielding effect and (iii) examine the potential correlation between the femur length and the femoral stem length, which exhibits optimal biomechanical behavior.

## MATERIALS AND METHODS

### Customized design

**Figure 1:**
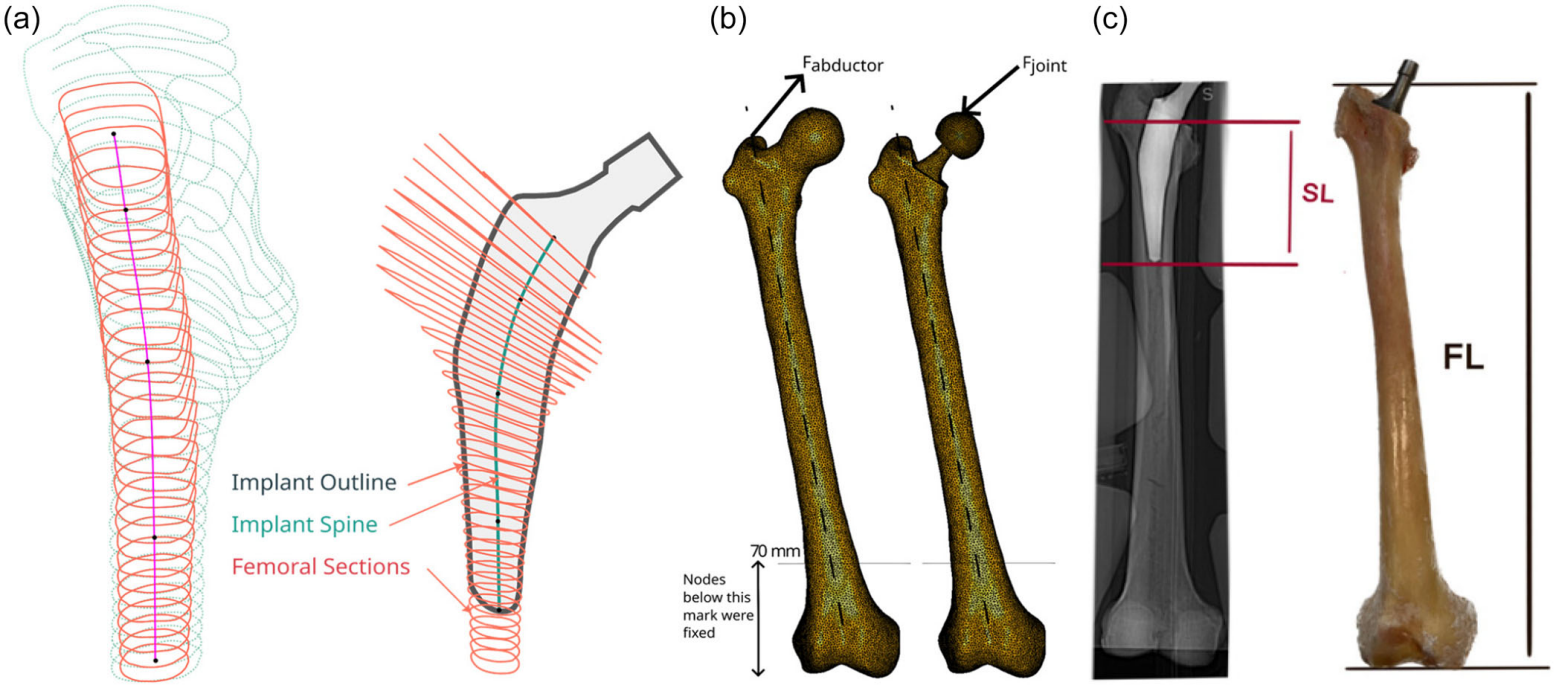
(a) Femoral cross‐sections and stem cross‐sections used in the design of custom‐made implants. Notice the implant spine that joins the centres of stem cross‐sections and whose length determines stem length. (b) The finite element model used for intact and implanted femurs shows mesh, loads, and boundary conditions. (c) Definitions of stem length (SL) and femur length (FL).

This study was designed as a computational FEA combined with an experimental cadaveric validation. Ethical approval for the cadaveric study was obtained from the Committee and Ethical Review Board of the University of Thessaly, Larissa (Acceptance Protocol Number 407). Inclusion criteria were complete coverage of the femoral head and distal condyles, and age >18 years. Exclusion criteria included: bone pathology as fracture or tumor presence and prior orthopedic implants. They were computed tomography (CT)‐scanned, using a momentary 90 kV, continuous 20 KV CT scanner model (HINOSHI). The scans were acquired with a voxel size of 0.6 × 0.6 × 1.25 mm^3^. For the fresh‐frozen cadaveric specimen, exclusion criteria included any femoral bone pathology or prior surgical intervention. The femur specimen (female, 72 years old, intact morphology, length 455 mm) was thawed at room temperature for 16 h and subsequently CT scanned (Figure 1). Preparation included careful removal of all surrounding soft tissues.

In total, 100 femoral models were reconstructed from anonymized clinical CT scans of adult lower limbs obtained from the institutional imaging database. These scans were used exclusively for virtual finite element simulations and did not correspond to cadaveric specimens. For experimental validation, one fresh‐frozen human cadaveric femur was selected. Exclusion criteria included any femoral pathology or prior surgical intervention. Femoral length was defined as the vertical distance between the highest point of the greater trochanter and the most distal point of the condyles (Figure 1c).

A senior orthopedic surgeon (280 procedures/year) used the custom hip implant design optimization service (CHIDOS) software (3Ψ Ltd., https://www.3-psi.com/) to design the patient‐specific implants. For each femur, a personalized and implantable femoral stem was designed and served as a base for the generation of 12 more stems, which were identical with the base design in all aspects apart from their length. This resulted in the simulation of 1300 stems in total. The stem lengths varied from each other with a step of 5 mm, being always within the range of 70–130 mm. Hence, for every femur 13 stems with lengths ranging from 70 to 130 mm in steps of 5 mm were studied. The stem length was measured from the level of the osteotomy on the medial region down to the stem's lowest tip (Figure 1c). Initially, the CT scans are processed to accomplish two primary tasks: segmentation of femoral and pelvic bones and localization of anatomical landmarks (condyles, isthmuses, trochanters and head center). For both tasks, CHIDOS employs the same Neural Network Architecture (NN), based on UNet Transformers (UNETR). The implant design procedure requires accurate representations of anatomical structures in the form of three‐dimensional (3D) tri‐meshes. To this end, 3D meshes were generated from the binary NN output masks using the marching cubes algorithm. This method allows converting the segmented volumes into precise triangulated mesh representations of the femoral and pelvic bones. Following the mesh generation, each femoral bone is digitally resected to emulate the surgical resection process, with user‐specific resection planes. The resected femoral mesh is then sliced, with a fixed spacing of 3 mm, to produce planar sections from both the periosteal and endocortical surfaces. The precise locations of the anatomical landmarks are derived by processing the binary label masks resulting from the NNs.

The final implant's shape is determined as follows: target values for anteversion, neck shaft and implant offset are provided by the user and utilized to parametrize the fixed‐shaped extramedullary portion of the implant. The algorithm automatically designs the intramedullary portion of the implant by creating planar profiles below the osteotomy level. They are restricted to be coplanar with the endocortical sections and confined within their boundaries, while at the same time, their shape depends on the exact locations of the anatomical landmarks. The final implant is created by forming a base mesh from those profiles (Figure 1a).

### Finite element model (FEM)

For each femur, an FEA of both the intact and the implanted bone models was performed. The loading configuration followed the standardization method suggested by Cristofolini and Viceconti [11]. The models were aligned to the reference system as suggested by Ruff and Hayes [33]. A joint force of 2.47BW (body weight) at 29° to the femoral diaphysis was applied over a patch, which was defined by the nodes being in a radius of 5 mm from the highest point of either the intact bone's femoral head or the implant's sphere (Figure 1b). For all cases, an abductor muscle's force of 1.55BW at 40° to the femoral diaphysis was distributed over a patch, which was defined by the nodes being in a radius of 5 mm from the highest node of the bone at the greater trochanter area. Both loads were applied statically and monotonically. The nodes of the lowest 70 mm of the femur were rigidly clamped, prohibiting the model to move in any direction. The femoral bone has a cortical shell and a cancellous core, each of which has different properties. Orthotropic material properties were assigned for both cancellous and cortical bone and a linear elastic behavior was assumed throughout loading [4, 8, 10]. Table 1 summarizes the assigned FEM properties. A detailed description of the FE model is provided as Supporting Information.

**Table 1:** Model parameters used in FEA [1, 3, 12, 13, 19, 21, 25, 29, 34, 42, 43, 44].

Parameter	Value		
Element type	First‐order (linear) tetrahedral elements (C3D4)		
Edge length	2 mm		
Joint force	2.47BW		
Abductor force	1.55BW		
Friction coefficient	0.3		
Contact type	Nonlinear frictional contact using face‐to‐face penalty algorithm		
Contact stiffness	50.000 N/mm^3^		

**Material**	**Elastic modulus (GPa)**	**Shear modulus (GPa)**	**Poisson's ratio (ν)**
Cortical bone	*E*_11_ = *E*_22_ = 11.55, *E*_33_ = 17	*G*_12_ = 3.6, *G*_13_ = *G*_23_ = 3.3	*v*_12_ = 0.58, *v*_13_ = *v*_23_ = 0.31
Trabecular bone	*E*_11_ = *E*_22_ = 4, *E*_33_ = 6.6	*G*_12_ = 2.8, *G*_13_ = *G*_23_ = 4.8	*v*_12_ = 0.4, *v*_13_ = *v*_23_ = 0.25
Titanium alloy	110	—	0.31

Abbreviations: BW, body weight; FEA, finite element analysis.

### Experimental validation

Τhe FEM was validated through a cadaveric study, approved by the Committee and Ethical Review Board of the University of Thessaly, Larissa (Αcceptance Protocol Number 407). For the cadaveric specimen, a customized stem was manufactured via Atomic Diffusion Additive Manufacturing (ADAM) in the in‐house MetalX 3D Printer (Markforged). The same material and manufacturing parameters were employed to produce a set of three custom broaches and one custom osteotomy guide. Following manufacturing, all devices were cleaned into Markforged Wash‐1, a solvent‐based debinding system and were sintered into Markforged Sinter‐1, a tube furnace.

The senior surgeon, a member of the design group of the implants in CHIDOS software, performed the implantation of the femoral stem into the cadaveric femur with the aid of the respective osteotomy guide and broaches. The implanted specimen was CT‐scanned, to compare the actual bone–implant system with the 3D model, and was refrozen. It was again thawed out at room temperature approximately 16 h before the experiment. Then, approximately 70 mm of the specimen's distal extremity was cemented using PALACOS® R radiopaque, poly(methyl methacrylate)‐based (PMMA) bone cement (Heraeus), into a rectangular thermoplastic pot, at 0° flexion in the sagittal plane and 11° adduction in the coronal plane [11]. A 36 mm Biolox Delta femoral head (CeramTec) was assembled on the stem. Subsequently, the anterior, medial and lateral surfaces of the proximal femur and the extramedullary stem were sprayed using matte white color and left to dry. Finally, a high contrast, random speckle pattern was created using matte black spray.

**Figure 2:**
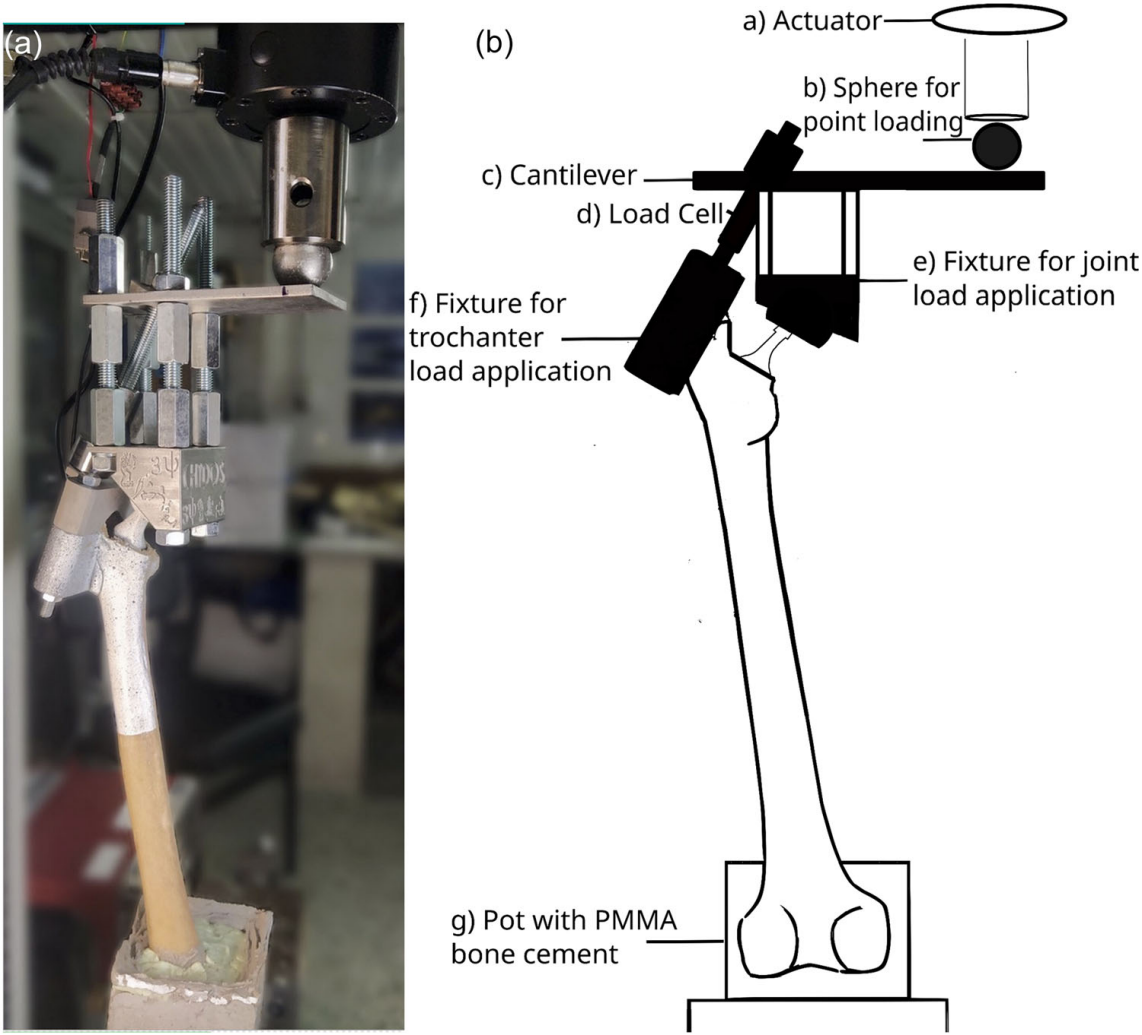
(a) Custom‐made apparatus simulating the loading configuration on the implanted cadaveric specimen as proposed by Cristofolini and Viceconti [11]. (b) Schematic representation of the apparatus showing (a) Actuator and load cell of the MTS machine, (b) Metallic sphere used for point loading, (c) Cantilever upon which everything is fixed, (d) Load cell to monitor abductor force, (e) Fixture with spherical cavity for load application on femoral head, (f) Fixture encapsulating Greater Trochanter for load application, (g) Pot in which the femur is fixed using PMMA. PMMA, poly(methyl methacrylate).

A custom‐made apparatus (Figure 2) was developed to simulate the quasi‐static loading configuration proposed by Cristofolini and Viceconti [11]. The experiment was carried out using an MTS Insight 10 kN electromechanical testing machine. The specimen was loaded 10 times, and average displacements over 10 repetitions were calculated. Maximum force was held for 180 s, and the specimen was allowed to recover for 60 s between repetitions. Digital image correlation (DIC) technique was used to record the cortical surface displacement distribution in the proximal, anterior and mediolateral area of the specimen. The 3D‐DIC system used (LIMESS Messtechnik & software GmbH) comprised two white light sources and two digital cameras. The resolution of the cameras was equal to 1624 × 1234 pixels, and the measuring field of view was 192 × 102 mm^2^. The accuracy for displacement measurements was equal to 0.01 pixel. Before the experiment, the system was calibrated with the aid of a suitable calibration target by Dantec Dynamics GmbH, Skovlunde, Denmark. A sampling rate equal to one frame per second was set. The obtained images were analyzed using the Istra4D v.4.2.3 software (Dantec Dynamics GmbH).

### Selection criterion regarding the best stem length per patient

Towards the formulation of a criterion regarding the selection of the optimal stem length per patient, the stress shielding effect and the developed micromotion for the different lengths were simultaneously evaluated. The selection criterion was defined as the minimum arithmetic mean of the normalized stress shielding score (sss) and the normalized micromotion, considering an equivalent contribution of each performance factor.

#### Normalized sss

**Figure 3:**
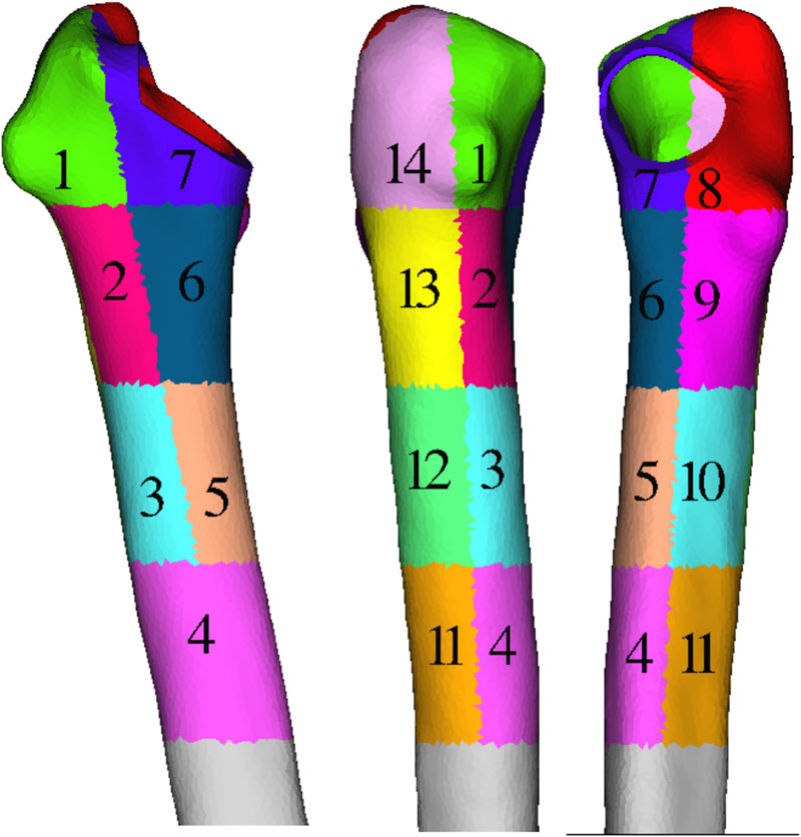
Division of the femur in 14 zones for the *sss* evaluation. sss, stress shielding score.

According to the classification proposed by Gruen [17], every femur was divided into 14 zones (Figure 3) using the femur axis as the dividing line between anterior‐posterior and proximal‐lateral parts. The stresses were obtained in all 14 zones, and a normalized sss for every stem length was calculated having the intact femur as reference. More specifically, for each Gruen zone, the difference of the mean von Mises stresses between the intact and the implanted cortical bones over the von Mises stresses of the intact femur was calculated, and the average of these normalized differences for all 14 zones was defined as the *sss*, according to the following equation:

(1) $$\mathrm{sss}=\Sigma \left(\frac{\sigma_{v\mathrm{Mises}}^{\mathrm{intact}}-{\;\sigma }_{v\mathrm{Mises}}^{\mathrm{implanted}}}{\sigma_{v\mathrm{Mises}}^{\mathrm{intact}}}\right)/14.$$

#### Normalized micromotion

Micromotion was measured for all stem's surface nodes between the osteotomy level and 20 mm below the Lesser Trochanter. For these implant nodes, the closest femoral nodes were detected, and their relative displacement was calculated. A min‐max normalization was applied to the mean micromotion value for every implant, after setting the acceptable range for micromotion as 15–150 μm [36]. The ratio (scaled to this range) of the micromotion measured to the maximum acceptable micromotion (150 μm) was defined as the normalized micromotion score. Hence, for a measured micromotion of, for example, 60 μm, the normalized micromotion score is 60–15/150–15 = 0.33. Values out of this range were assigned a score of 1.

### Statistical analysis

Linear regression analysis was performed using R software (R Foundation for Statistical Computing, Vienna, Austria, RRID:SCR_001905, Version 2023.03.0 + 386) to assess the effect of stem‐to‐femur length ratio on the two biomechanical performance indicators: micromotion and sss. The following separate linear regression models were formulated: Micromotion_i_ and sss_i_ are the normalized micromotion and sss for each stem length *i*, *β*₀ is the intercept, *β*₁ is the slope coefficient estimating the effect of the stem‐to‐femur length ratio and *ε*_i_ is the residual error term, assumed to follow a normal distribution (*ε* ~ N(0, σ²)).

**Model 1:** Micromotion_i_ = *β* + *β*₁ × (Stem‐to‐Femur Length Ratio)_i_ + *ε*_i_.

**Model 2:** sss_i_ = *β*₀ + *β*₁ × (Stem‐to‐Femur Length Ratio)_i_ + *ε*_i._

Both dependent variables were previously normalized as described in Section Selection criterion regarding the best stem length per patient. Assumptions of normality (verified via the Shapiro–Wilk test), linearity and homoscedasticity were confirmed. A *p*‐value threshold of 0.05 was used for statistical significance.

## RESULTS

### FEM validation

**Figure 4:**
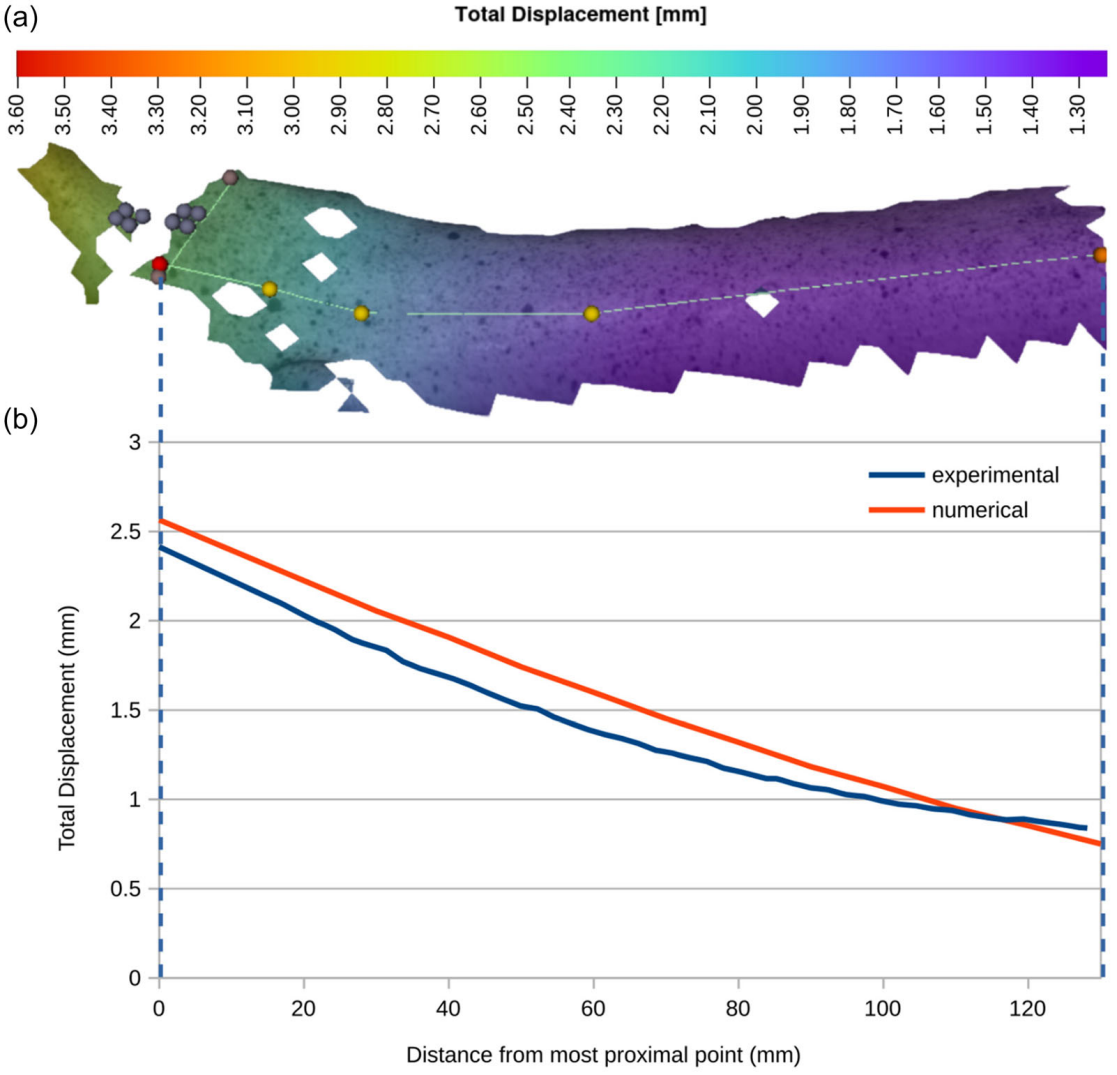
(a) Total displacement of the cadaveric specimen as measured by digital image correlation (DIC) (the red and yellow dots are used to bound the start, the end and the midpoints defining the sampling curve), (b) total displacement for a set of nodes on the anterior side of the cadaveric specimen as measured by the DIC (blue line) in comparison with the displacement calculated via the finite element analysis (red line) for the same locations on the finite element (FE) model.

DIC captured the displacement of 110 surface points on the anterior side of the implanted cadaveric specimen throughout the static loading (Figure 4a illustrates some of these points). Both experimental and numerical displacements in the three dimensions were calculated for 14 of these points, and the graph of Figure 4b depicts the difference of total displacement values as obtained from DIC and FEA. The difference was found to be 0.17 mm ± 0.09 mm (mean, SD) in which the x‐axis of the graph represents the different points based on their distance from the most proximal one (red‐colored dot in Figure 4a). The agreement between the experimentally measured and the numerically predicted displacements is considered quite satisfactory; hence, the validated FEM was subsequently used to examine the stem length's influence on the biomechanical behavior of the implants.

### FEA results statistical analysis

**Figure 5:**
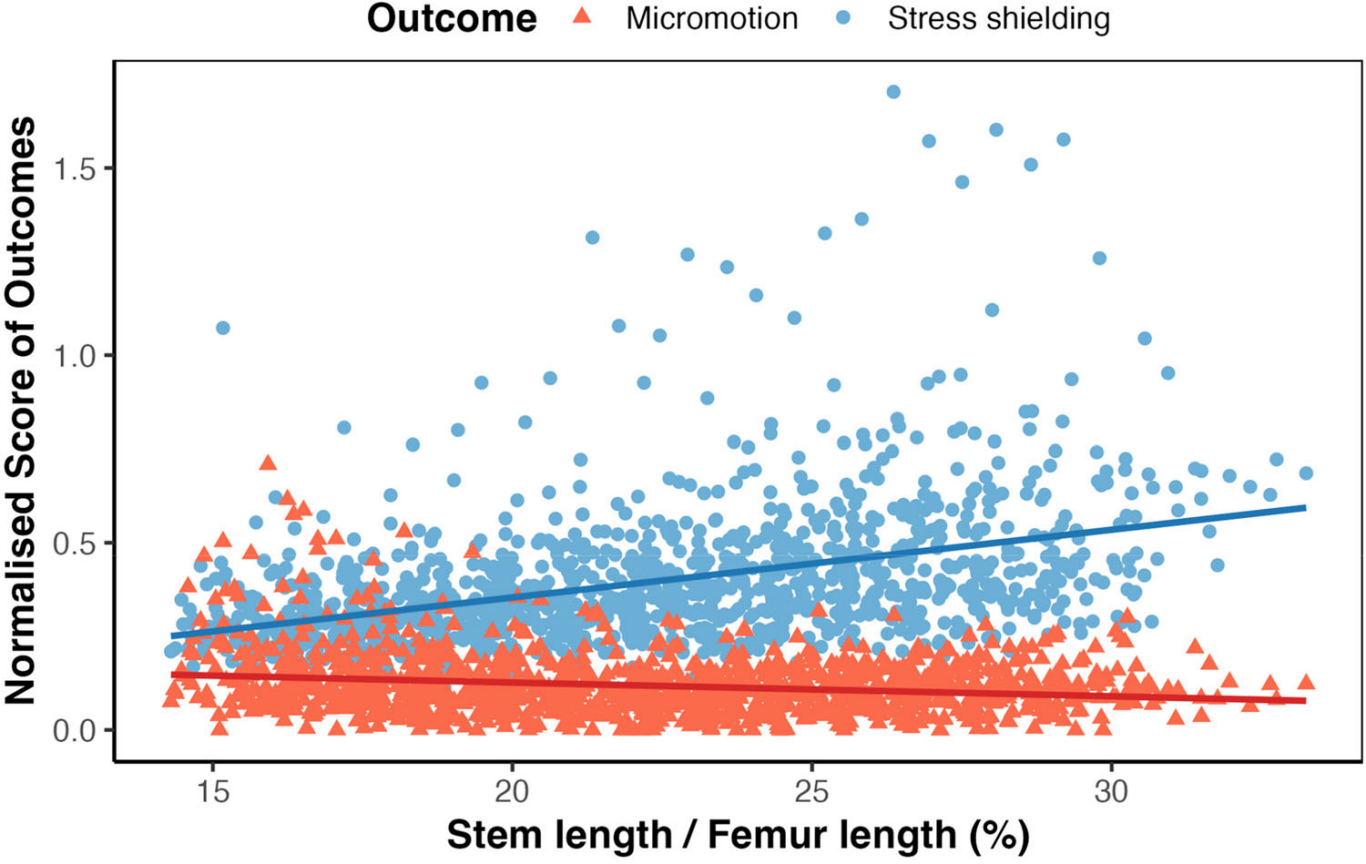
Linear regression analysis of stress shielding and micromotion versus stem‐to‐femur length ratio.

A normal distribution for all variables was confirmed via the Shapiro–Wilk Test. The analysis, which is presented in Table 2 and Figure 5, revealed a stress shielding coefficient of 1.811 (Standard Error = 0.114, *p* = 2 × 10^−16^) explaining approximately 17.2% of the variance (*R²* = 0.172) and a micromotion coefficient of –0.367 (standard error = 0.054, *p* = 1.14 × 10^–11^) accounting for 3.7% of the variance (*R²* = 0.037), indicating that for each unit increase in the fraction of stem‐to‐femur length, the stress shielding increases by 1.811 while the micromotion decreases by 0.367. For both factors, *p*‐values denote a significant and inverse effect of the stem length in the developed micromotion and stress shielding effect. Further information is presented in Table 2; the predictor is the stem‐to‐femur length ratio, and the estimate (β) represents how much the outcome variable changes for each unit increase in this ratio. For example, the estimate of –0.367 for normalized micromotion score indicates that greater stem‐to‐femur ratios are associated with reduced micromotion at the bone–implant interface. Conversely, the estimate of +1.811 for stress shielding shows that longer stems increase the degree of proximal stress shielding. The *t* value is the test assessing whether the effect differs significantly from zero; higher absolute *t* values indicate stronger evidence of an effect. Both regression models demonstrated highly significant results (*p* < 0.001), confirming that stem‐to‐femur ratio significantly influences both micromotion and stress shielding, but in opposite directions.

**Table 2:** Linear regression results for stress shielding and micromotion.

Outcome Variable	Predictor	Estimate (β)	Std. error	*t* value	*p* value	*R*²
Normalized micromotion (score)	Intercept	0.200	0.012	16.44	<0.0001***	
	Stem length	–0.367	0.054	–6.85	<0.0001***	0.037
Normalized Stress shielding score (score)	Intercept	–0.008	0.026	–0.33	0.745	
	Stem length	+1.811	0.114	+15.95	<0.0001***	0.172

*** *p* < 0.001, residual SD for micromotion = 0.080; for score = 0.169, *n* = 1228 observations.

**Figure 6:**
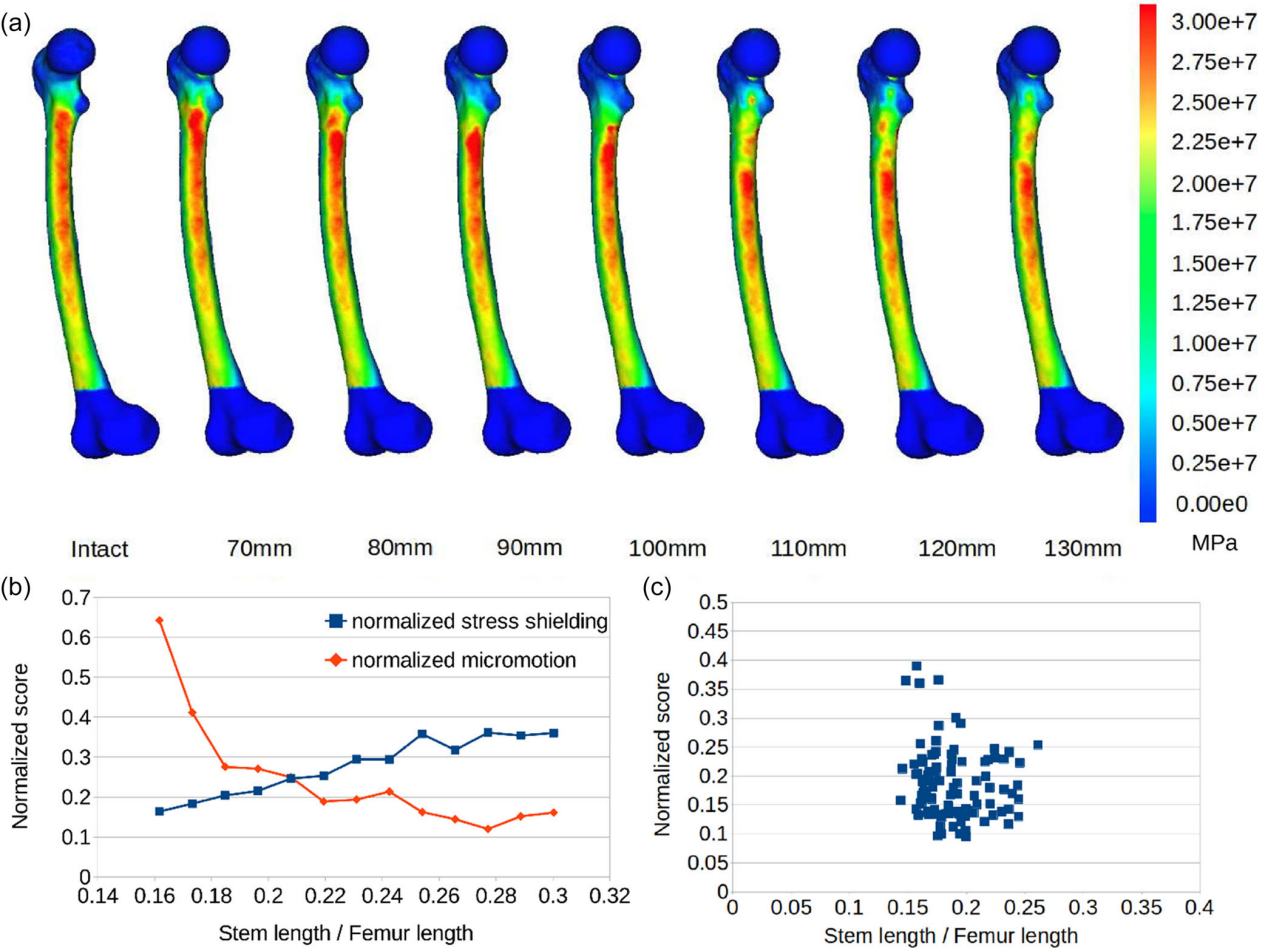
(a) von Mises stress distribution on intact and implanted femurs for stem lengths ranging from 70 mm to 130 mm for a single specimen, (b) normalized micromotion and normalized *sss* against stem‐to‐femur length ratio for the same specimen, (c) dispersion of optimal femoral stem lengths (fraction of total femur length) in terms of the arithmetic mean of normalized micromotion and sss computed for all cases. Each dot represents the minimum of this value of each case for all 100 cases. sss, stress shielding score.

Figures 6a,b comprise a characteristic example of the 100 cases; normalized micromotion and *sss* were simultaneously plotted in a single graph, in which the *y*‐axis represents both normalized scores and the *x*‐axis represents the different stem‐to‐femur length ratios for this specific case (Figure 6b). These figures indicate a stress shielding effect deterioration with stem lengthening, whereas a smaller differentiation in micromotion values for the different lengths can be observed in Figure 6b. Calculated micromotion was overall very low (28.5 μm, SD 6.75 μm) regardless of the stem length. In order to assess the performance of each stem, the arithmetic mean of the two performance factors was calculated as a single criterion. For each case, the optimal stem length is considered to be the one that minimizes this criterion.

The overall results suggest that lengthening the stem influences inversely the two performance indicators, with a more pronounced negative impact on the stress shielding effect, and the best trade‐off is achieved for a mean stem length of 18.5% (SD 2.7%) of the total femoral length.

## DISCUSSION

The results of this study verify the hypothesis that for the different lengths of the femoral bone, there is an optimal custom‐made stem length that achieves both excellent micromotion and considerable reduction of the stress shielding effect. A cadaveric study was carried out to validate an FEM, which was then applied to 100 femur cases. The results demonstrate that the stem length, which achieves both excellent micromotion and considerable reduction of the stress shielding effect, is 18.5% ± 2.7% (mean, SD) of the total femur length per case. In general, longer stems worsened the stress shielding effect, while the effect of stem length on micromotion was less pronounced apparently due to the tight fit of the personalized design. The shorter stems exhibit good contact and press‐fit with the surrounding bone, thus achieving good primary stability. In the case of noncustomized stems, shorter stems are assumed to develop higher micromotion [46]. While a recent study highlights that short and conventional uncemented stems in THA achieve equivalent implant survival [40], another study suggests that short metaphyseal stems provide better functional outcomes without subsidence, implant loosening or proximal femoral bone loss in a 15‐year follow‐up period [26]. Taken together, these data support our findings that the optimal stem‐to‐femur length ratio should be considered a crucial parameter for the appropriate femoral implant selection, together with the stem surface treatment, the coating technology as well as the quality of metaphyseal and diaphyseal implant‐to‐bone press‐fit.

Before concluding, some limitations should be mentioned. Bone remodelling and osseointegration are dynamic processes depending on several factors, like bone quality, surrounding soft tissue, patient lifestyle, and so on, which cannot be simulated in vitro [5, 15]. The validation of the FEM model should ideally be extended with more cadaveric specimens. Also, following the configuration of Cristofolini et al. [11], several muscle forces were excluded from the model due to their minor contribution to the developed micromotion according to previous studies [31]. Other loading configurations can also be investigated in an expanded study. An equivalent weighting factor for minimal micromotion and minimal stress shielding has been currently assumed, without, however, rejecting the possibility that unequal weights could be assigned to the two criteria, since micromotion lower than 150 μm is regarded as acceptable, while stress shielding should be minimized. Alternative weighting factors (Supporting Information) affect the optimum length, but determining their relative importance is challenging. Besides, the stress distribution differed between different intact bones, highlighting the diversity in human anatomy; hence, it should be anticipated that more selection criteria could be used, for example, bone stiffness [38]. We further acknowledge that proximal femoral morphology, characterized by Dorr classification, can significantly impact stem behavior and surgical outcomes. For instance, a lower survivorship rate (97.8% vs. 99.5%) and higher incidence of radiolucent lines and periprosthetic fracture was reported in Dorr type A femurs compared to type B when using a cementless tapered wedge stem [30]. Conversely, Zhen et al. [47] demonstrated that short, metaphyseal‐fitting cementless stems can achieve reliable stability in Dorr type C (stovepipe‑shaped) femurs, which often preclude conventional fixation. Our findings also align with recent systematic evidence demonstrating that implant design and fixation techniques strongly influence postoperative bone remodelling. A meta‐analysis of 68 clinical studies reported that cemented implants and tapered stems showed greater periprosthetic bone resorption than cementless implants and anatomical stems, particularly within the first 6 months after THA [2].

Other noteworthy characteristics that affect the biomechanical performance of the implant are material, design, geometry and surface treatment. For example, stem offset has been associated with aseptic loosening risk, with high offsets increasing the risk almost fourfold [23]. Moreover, implant fixation success depends on patient characteristics, bone quality, fixation strategy and surgical technique as well. Flexible stem designs and lower modulus alloys further mitigate stress shielding while preserving stability [18]. Additionally, porous or lattice structures—especially when additively manufactured—enable better load sharing and osseointegration, cutting bone loss by up to 75% [40]. Recent clinical evidence indicates hydroxyapatite (HA)–coated femoral stems have fewer stem revisions for aseptic loosening and less thigh pain than non‐HA stems, consistent with improved biological fixation [24, 34].

In terms of generalizability to off‐the‐shelf stems, our FEM framework evaluated personalized stems to isolate the effect of length while controlling other design variables. Although the length–response trends we observed are generic, direct extrapolation to all off‐the‐shelf designs should be cautious because commercial stems differ in proximal/distal geometry (metaphyseal vs. diaphyseal fit), taper and flare, collar, material stiffness and surface technology. These attributes influence canal fill, load transfer and osseointegration, and can shift the precise length that optimizes the trade‐off. Accordingly, we suggest that the stem‐to‐femur ratio should be interpreted as a biomechanical starting point that is most applicable to cementless, metaphyseal‐fitting press‐fit stems. In modular femoral systems, stem bodies of varying lengths can be combined with proximal sleeves or necks to tailor fixation. In such cases, our proposed stem‐to‐femur ratio could serve as a guide for selecting the appropriate stem body length, while modular proximal components could then be adjusted to optimize offset, anteversion and load transfer. However, modularity also introduces new biomechanical considerations, including potential stress concentrations at junctions and risks of fretting or corrosion, which were not simulated in our FEM.

With respect to cemented stems, our formula should not be directly extrapolated. Cemented fixation is based on a different principle, with the cement mantle altering load transfer, micromotion and stress shielding patterns. Indeed, a recent systematic review found greater bone resorption around cemented when compared with cementless stems [2]. For this reason, the optimal stem length in cemented arthroplasty must be investigated separately, and our findings are not universally applicable. Our findings should be interpreted within the context of an in vitro study, investigating the effects of the press fit implantation of the stem, specifically designed for each one of the tested femora.

Despite these limitations, the present study combines two key performance criteria (micromotion and stress shielding) and offers a simple tool that could be practically applied to aid the clinicians' practice by selecting the implant's length taking into account the patient's femur length. This work lays the foundation for correlating the appropriate implant size with anatomical and other variables. For customized designs, short stems seem to be the most effective solution; however, as each patient is unique, the surgeon's selection depends on multiple criteria besides stem biomechanics.

The researchers intend to expand the study in order to take into account standardized designs and investigate the impact of other anatomical parameters, aiming to assist orthopedic surgeons to provide partially patient‐specific treatment even in the case of standardized implants.

## CONCLUSION

This work demonstrated that longer femoral stems enhance initial stability and reduce micromotion, while shorter stems promote a more uniform stress distribution, potentially lowering the risk of aseptic loosening. Within the study limitations, the optimal stem length was determined to be 18.5 ± 2.7% of femur length. This results from a trade‐off between micromotion and stress shielding and indicates that personalized selection can optimize initial stability and hip implant biomechanics to better enhance osteointegration. Further research is required to verify those results in a clinical setting and investigate their validity for standardized stems.

## AUTHOR CONTRIBUTIONS

*Conceptualization*: Athanasios Batagiannis. *Data curation*: Georgios Banis, Ermioni Pasiou, Styliani Stergiadou. *Formal analysis*: Georgios Banis, Styliani Stergiadou. *Investigation*: Georgios Banis, Maria Sarkiri, Charoula Kousiatza, Ermioni Pasiou, Ilias Stavrakas, Konstantinos Kalkanis. *Methodology*: Maria Sarkiri, Charoula Kousiatza, Georgios Banis. *Project administration*: Athanasios Batagiannis. *Resources*: Styliani Stergiadou, Konstantinos Malizos, Michael Hantes, Aristeidis Zibis, Stavros Kourkoulis. *Software*: Κostas Touloupas, Alexis Spiliotopoulos, Panagiotis Kontopoulos. *Supervision*: Konstantinos Malizos, Stavros Kourkoulis, Ilias Stavrakas, Ermioni Pasiou, Konstantinos Kalkanis, Athanasios Batagiannis. *Validation*: Maria Sarkiri, Charoula Kousiatza, Ermioni Pasiou, Ilias Stavrakas, Konstantinos Kalkanis. *Visualization*: Alexis Spiliotopoulos, Georgios Banis. *Writing—original draft*: Maria Sarkiri, Georgios Banis, Charoula Kousiatza, Κostas Touloupas, Alexis Spiliotopoulos, Panagiotis Kontopoulos, Styliani Stergiadou. *Writing—review & editing*: Athanasios Batagiannis, Aristeidis Zibis, Michael Hantes, Konstantinos Malizos, Stavros Kourkoulis, Ermioni Pasiou, Ilias Stavrakas, Konstantinos Kalkanis.

## CONFLICT OF INTEREST STATEMENT

The authors declare no conflicts of interest.

## ETHICS STATEMENT

The study was approved by the Ethical Review Board of University of Thessaly, Larissa (Αcceptance Protocol Number: 407).

## Supporting information


**Figure S1.** a) Max displacement and von Mises stress as a function of mesh element size. b) Max micromotion and von Mises stress as a function of contact stiffness. **Figure S2.** Dispersion of optimal femoral stem lengths (% of total femur length) computed for all cases under three different weighting schemes of the performance factors: equal weighting between stress shielding score (sss) and micromotion (1:1, shown in blue), stress shielding weighted twice as heavily as micromotion (2:1, shown in yellow), and micromotion weighted twice as heavily as stress shielding (1:2, shown in red). Each point represents the optimal stem length for an individual case based on the specified weighting and illustrates the sensitivity of the optimal length to the selected trade‐off between micromotion and stress shielding.

## Data Availability

All data are available upon request by the author.
