# Supplementary material for: Does the ratio of stem‐to‐femur length matter? A validated finite element analysis
Source: J Exp Orthop. 2025 Oct 31;12(4):e70484. doi: 10.1002/jeo2.70484 (PMC12578472; doi:10.1002/jeo2.70484)
Supplement: Supplementary file 1 — Figure S1. a) Max displacement and von Mises stress as a function of mesh element size. b) Max micromotion and von Mises stress as a function of contact stiffness. Figure S2. Dispersion of optimal femoral stem lengths (% of total femur length) computed for all cases under three different weighting schemes of the performance factors: equal weighting between stress shielding score (sss) and micromotion (1:1, shown in blue), stress shielding weighted twice as heavily as micromotion (2:1, shown in yellow), and micromotion weighted twice as heavily as stress shielding (1:2, shown in red). Each point represents the optimal stem length for an individual case based on the specified weighting and illustrates the sensitivity of the optimal length to the selected trade‐off between micromotion and stress shielding. [file JEO2-12-e70484-s001.docx]

**Supplementary Material**

**FEA model detailed description**

Both femurs and respective stem models were meshed in GMSH Version 4.12.1 (RRID:SCR_021226, <https://gmsh.info/>) using first-order (linear) tetrahedral elements (C3D4) [[4, 42]](https://www.zotero.org/google-docs/?DKHTQO). A fine mesh was chosen, in order to obtain results of sufficient accuracy, using element sizes of maximum 2 mm edge length.

The femoral bone has a cortical shell and a cancellous core, each of which has different properties. Orthotropic material properties were assigned for both cancellous and cortical bone and a linear elastic behavior was assumed throughout loading [[3, 8, 43]](https://www.zotero.org/google-docs/?juvTNP) A sphere having the same center and diameter with the intact femoral head was designed for each femur and fused with the stem, in order to together comprise a single body, since its deformations are considered negligible. The sphere was added as a simplified representation of the THA ball and acetabular cup and was used so that the joint force application would be the same as that of the intact femur (Figure 1b).

Surface remeshing was implemented using Meshlab software, v2023.12 (RRID:SCR_027065, [https://www.meshlab.net)](https://www.meshlab.net/), by performing explicit isotropic remeshing to regularize size and aspect ratio of the triangular mesh [10]. A mesh sensitivity test was conducted to ensure the accuracy and convergence of the finite element model. The study assessed the influence of element edge length on the maximum displacement and the maximum vonMises stress (Figures S1a and S1b). Meshes in a range from 1-10 mm were generated and the results indicated that element sizes larger than 2 mm produced variations in both displacement and stress values, suggesting insufficient resolution. Mesh refinements below 2 mm for the bone and 1.5 mm for the implant and trabecular resulted in minimal changes in the evaluated outputs while increasing the computational cost.

The bone-stem interface was simulated based on a non-linear frictional contact model, using the face-to-face penalty formulation algorithm of CalculiX Version 2.2 (RRID:SCR_024771, <https://www.calculix.de/>). It was assumed that after THA, during the period in which osseointegration has not been initiated, bone and implant are not fully bonded. An initial tangential sliding and a frictional coefficient μ=0.3 were considered until contact was achieved [[1, 29, 44]](https://www.zotero.org/google-docs/?j7w1ww). In regions where contact was expected, a normal contact was defined with separation (no bone ongrowth was assumed in the first weeks after implantation). A contact sensitivity analysis was performed to determine an appropriate penalty stiffness value for the bone-implant interface. A series of simulations was conducted with contact stiffness values ranging from 10^3^ to 10^7^ N/mm^3^. The results showed that maximum vonMises stresses displayed numerical instability for stiffness values exceeding 50.000 N/mm^3^, indicating over-constraining of the contact interface. In contrast, micromotion decreased with increasing stiffness and reached a plateau for values above 50.000 N/mm^3^, suggesting convergence. Based on this trade-off, a contact stiffness of 50.000 N/mm^3^ was chosen to specify the contact pressure-overclosure relationship.


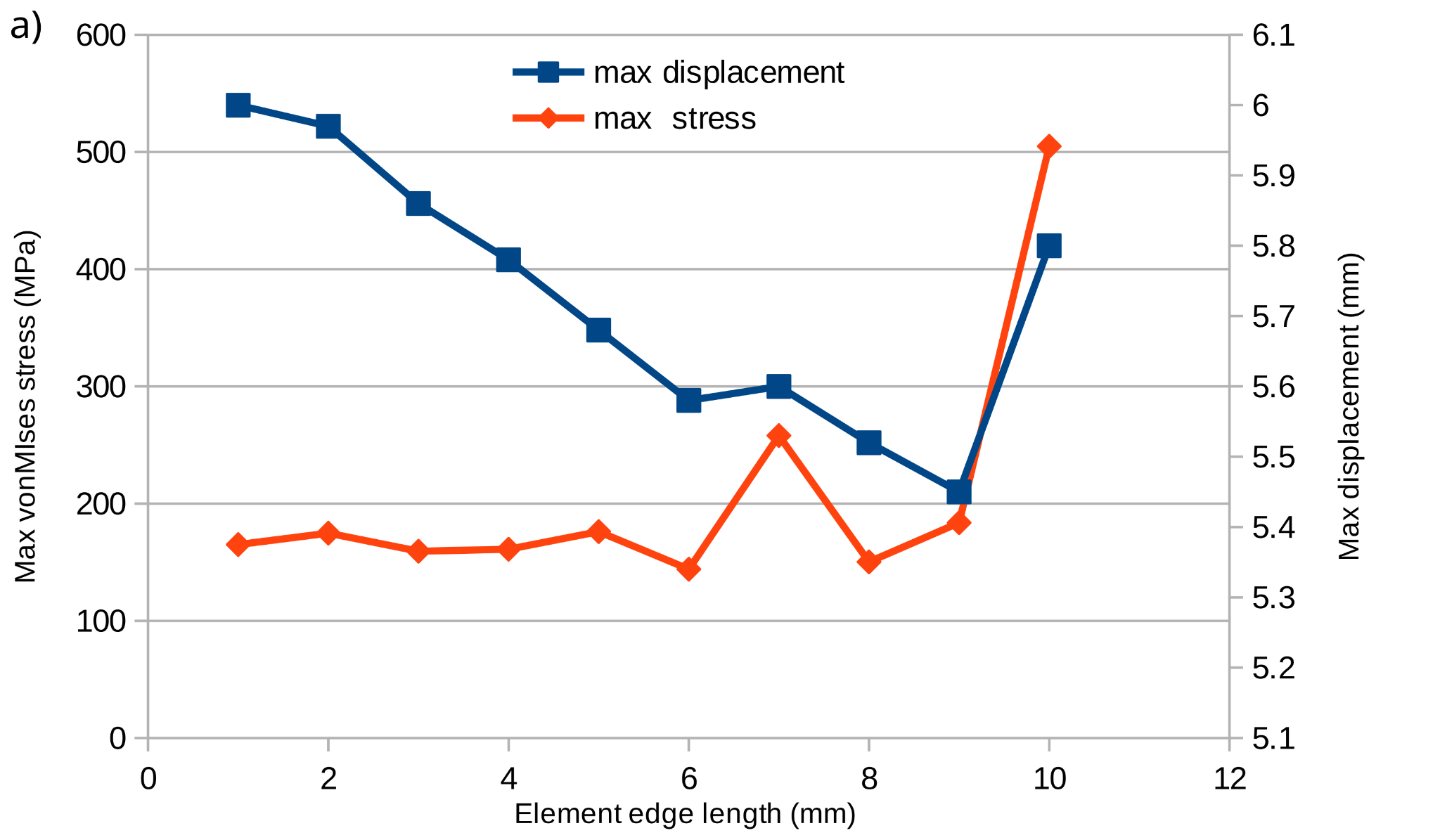

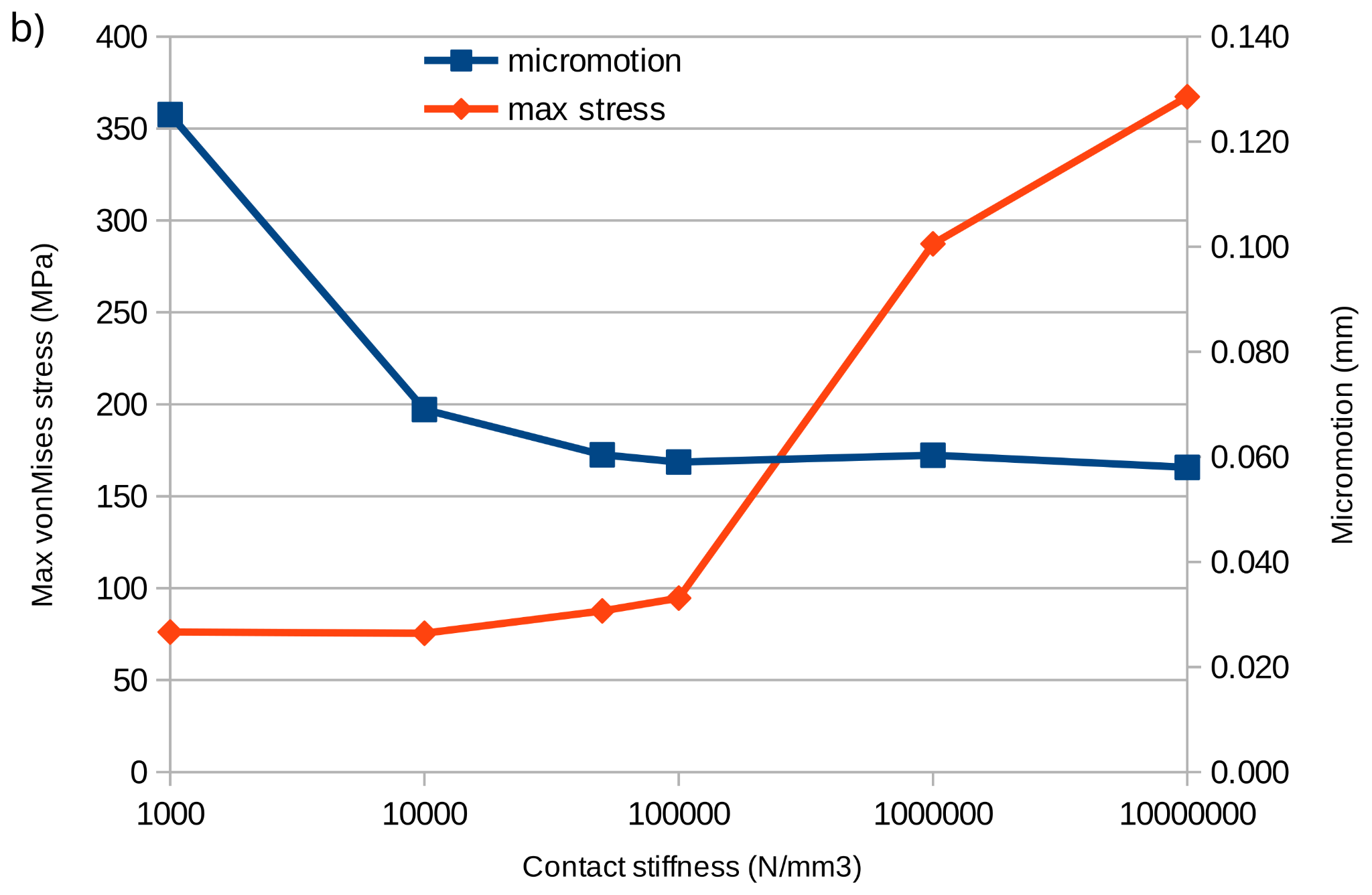


**Figure S1.** a) Max displacement and von Mises stress as a function of mesh element size. b) Max micromotion and von Mises stress as a function of contact stiffness.

**Effect of weighting factor on normalized score**

To assess the sensitivity of the optimal stem length selection to the assumed equal weighting between stress shielding score (sss) and micromotion, additional analyses were conducted using two alternative weighting schemes: (i) stress shielding weighted twice as heavily as micromotion (2:1), and (ii) micromotion weighted twice as heavily as stress shielding (1:2). Under the 2:1 weighting, the mean optimal stem length was calculated as 18.15% (standard deviation = 2.4%) of the total femoral length. Conversely, the 1:2 weighting yielded a slightly higher mean optimal stem length of 19.3% (standard deviation = 3.4%). These results indicate that while the precise optimal length is somewhat sensitive to the weighting between performance criteria, the overall range and trend remain consistent with the original analysis. The revised dispersion plots corresponding to these two additional weighting scenarios are shown in Supplementary Figure S2, overlaid on the original data for comparison.


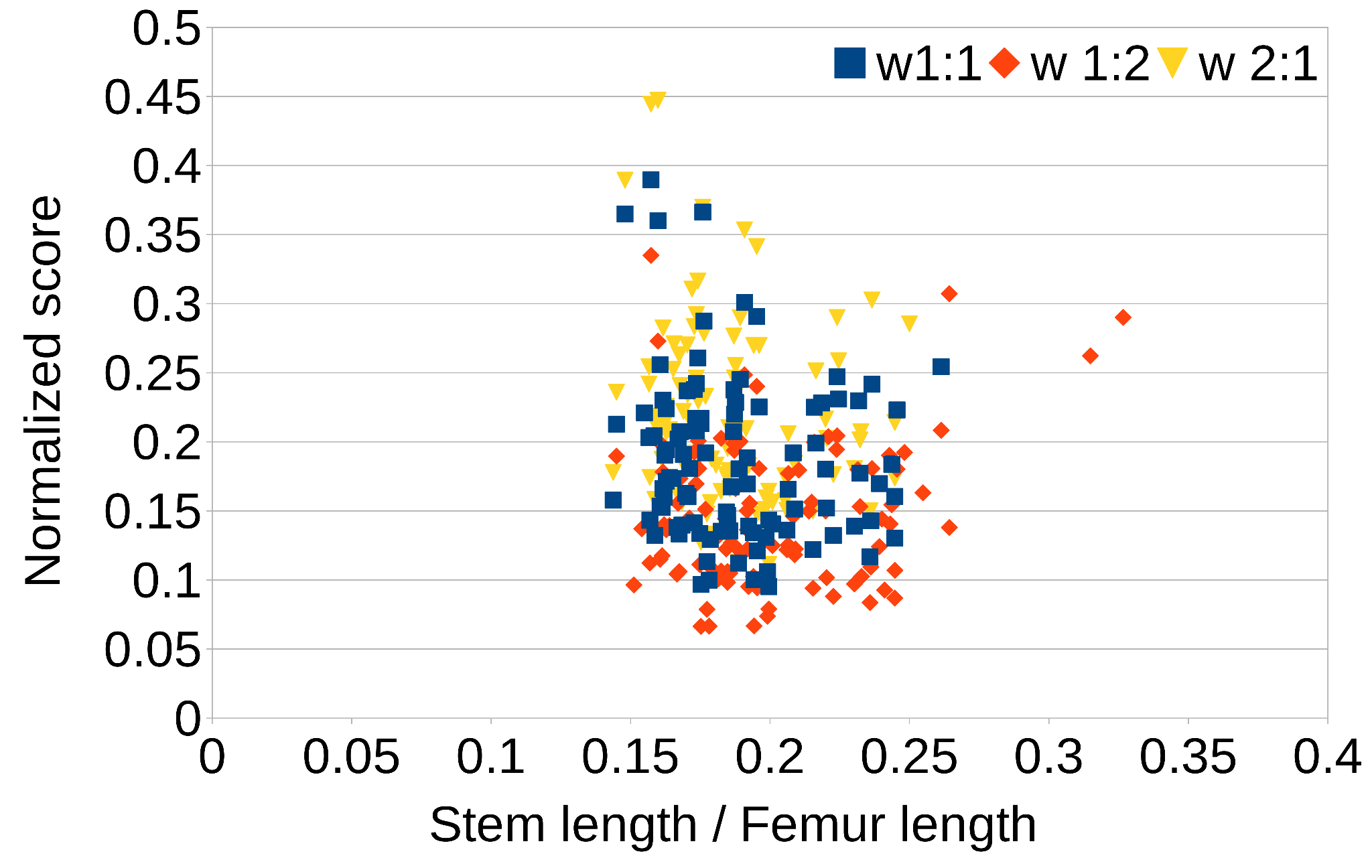


**Figure S2.** Dispersion of optimal femoral stem lengths (% of total femur length) computed for all cases under three different weighting schemes of the performance factors: equal weighting between stress shielding score (sss) and micromotion (1:1, shown in blue), stress shielding weighted twice as heavily as micromotion (2:1, shown in yellow), and micromotion weighted twice as heavily as stress shielding (1:2, shown in red). Each point represents the optimal stem length for an individual case based on the specified weighting and illustrates the sensitivity of the optimal length to the selected trade-off between micromotion and stress shielding.

**List of Figures/Tables:**

**Figure S1.** a) Max displacement and von Mises stress as a function of mesh element size. b) Max micromotion and von Mises stress as a function of contact stiffness.

**Figure S2.** Dispersion of optimal femoral stem lengths (% of total femur length) computed for all cases under three different weighting schemes of the performance factors: equal weighting between stress shielding score (sss) and micromotion (1:1, shown in blue), stress shielding weighted twice as heavily as micromotion (2:1, shown in yellow), and micromotion weighted twice as heavily as stress shielding (1:2, shown in red). Each point represents the optimal stem length for an individual case based on the specified weighting and illustrates the sensitivity of the optimal length to the selected trade-off between micromotion and stress shielding.
